# Lack of Toxicity in Nonhuman Primates Receiving Clinically Relevant Doses of an AAV9.U7snRNA Vector Designed to Induce *DMD* Exon 2 Skipping

**DOI:** 10.1089/hum.2020.286

**Published:** 2021-09-23

**Authors:** Liubov V. Gushchina, Emma C. Frair, Natalie Rohan, Adrienne J. Bradley, Tabatha R. Simmons, Hemantkumar D. Chavan, Hsin-Jung Chou, Michelle Eggers, Megan A. Waldrop, Nicolas Wein, Kevin M. Flanigan

**Affiliations:** ^1^ The Center for Gene Therapy, Abigail Wexner Research Institute, Nationwide Children's Hospital.; ^2^ Charles River Laboratories, Wilmington, Massachusetts, USA.; ^3^ Audentes Therapeutics, San Francisco, California, USA.; ^4^ Department of Pediatrics, The Ohio State University, Columbus, Ohio, USA.; ^5^ Department of Neurology, The Ohio State University, Columbus, Ohio, USA.

**Keywords:** Duchenne muscular dystrophy, adeno-associated virus, noncoding U7snRNA

## Abstract

Therapeutic exon skipping as a treatment for Duchenne muscular dystrophy (DMD) has largely concentrated on the delivery of antisense oligomers to treat out-of-frame exon deletions. Here we report on the preclinical development of an adeno-associated virus (AAV)-encapsidated viral vector containing four copies of the noncoding U7 small nuclear RNA (U7snRNA), each targeted to either the splice donor or the splice acceptor sites of *DMD* exon 2. We have previously shown that delivery of this vector (scAAV9.U7.ACCA) to the Dup2 mouse model results in expression of full-length dystrophin from wild-type *DMD* mRNA, as well as an internal ribosome entry site (IRES)-driven isoform translated only in the absence of exon 2 (deletion exon 2 [Del2] mRNA). Here we present the data from a rigorous dose escalation toxicity study in nonhuman primates, encompassing two doses (3 × 10^13^ and 8 × 10^13^ vg/kg) and two time points (3 and 6 months postinjection). No evidence for significant toxicity was seen by biochemical, histopathologic, or clinical measures, providing evidence for safety that led to initiation of a first-in-human clinical trial.

## Introduction

Therapeutic restoration of an open reading frame by selectively excluding exons from a mature mRNA transcript, or exon skipping, is an approach that is already in clinical use for a subset of patients with Duchenne muscular dystrophy (DMD).^1^ Currently approved therapies use repeated intravenous infusions of antisense oligomers complementary to target exons of interest adjacent to out-of-frame exon deletions, restoring an open mRNA reading frame and allowing translation of internally deleted but partially functional proteins. An alternative viral vector-based approach uses a noncoding U7snRNA modified with a terminal sequence targeting exon. As a result, the single-stranded U7snRNA, stabilized by interaction with the SmOpt protein, binds to the pre-mRNA, resulting in the exclusion of this exon.^2–7^ This approach is likely to be advantageous in that expression of U7snRNA from the adeno-associated virus (AAV) genome and subsequent exon skipping is long-standing,^8^ which may allow a single treatment rather than weekly infusions.

We have developed such a viral vector carrying sequences targeting exon 2 within a self-complementary (sc) AAV genome. Our vector, scAAV9.U7.ACCA, carries four copies of U7snRNA, with two directed toward the splice acceptor sequence (sequence A) and two targeting the splice donor sequence (sequence C) encapsidated within AAV9. *In silico* analysis has shown these sequences to be specific to the intron/exon junctions for human exon 2, and the targeted sequences are 100% identical between human and nonhuman primates (NHPs). We have developed this vector intending to initially target not out-of-frame deletions, but instead single-exon duplications in the *DMD* gene. In contrast to the case with deletions, skipping of one of the two duplicated exons would restore an entirely wild-type (WT) mRNA and expression of full-length dystrophin. We chose exon 2 for proof-of-concept for two reasons. First, it is the most common single-exon duplication seen in DMD patients.^9,10^ Second, skipping of exon 2 provides an enormous therapeutic window. As we have previously demonstrated in a novel mouse model carrying an exon 2 duplication,^11^ deletion of both copies of exon 2 results in the utilization of an internal ribosome entry site (IRES) within exon 5, resulting in translation of a highly functional nearly full-length dystrophin protein that may allow ambulation into the eighth decade.^12–14^ Therefore, complete exclusion (or “overskipping”) that reaches a deletion exon 2 (Del2) transcript is still therapeutic.

The objective of this study was to determine the potential toxicity of the scAAV9.U7.ACCA vector in NHPs. The study was performed as part of a preclinical development plan that led to the initiation of an ongoing gene therapy trial using the vector we describe (ClinicalTrials.gov NCT04240314).

## Results

Before enrollment, male juvenile cynomolgus monkeys were screened for circulating antibody titers to AAV9 capsid determined by enzyme-linked immunosorbent assay (ELISA); all were seronegative (titer <1:5). Nine animals were enrolled and dosed with either diluent (*n* = 3), a low dose (3 × 10^13^ vg/kg, *n* = 3), or higher dose (8 × 10^13^ vg/kg, *n* = 3) of scAAV9.U7.ACCA (Table 1), doses determined following dose escalation studies to establish the minimal efficacious dose planned for a human clinical trial (data not shown).

**Table 1.** Experimental design

Group No.	Test Material	Dose Level (vg/kg)	Dose Volume (mL/kg)	Dose Concentration (vg/mL)	No. of Males	
					3 Months	6 Months
1	Control article	0	8	0	2	1
2	scAAV9.U7.ACCA	3 × 10^13^	8	0.37 × 10^13^	2	1
3	scAAV9.U7.ACCA	8 × 10^13^	8	1 × 10^13^	2	1

### Clinical assessments

Clinical assessments occurred throughout the study period, and included clinical signs, body weights, qualitative food consumption, and neurological examinations. Ophthalmologic examination and echocardiograms were performed before test article delivery and again in the week before necropsy. All animals tolerated the injections well, with no acute reactions to the test article at either dose. Over the entire period of the study, no clinical observations were reported that suggested toxicity.

### Biochemical, hematological, and immunological assessments

**Figure 1.**
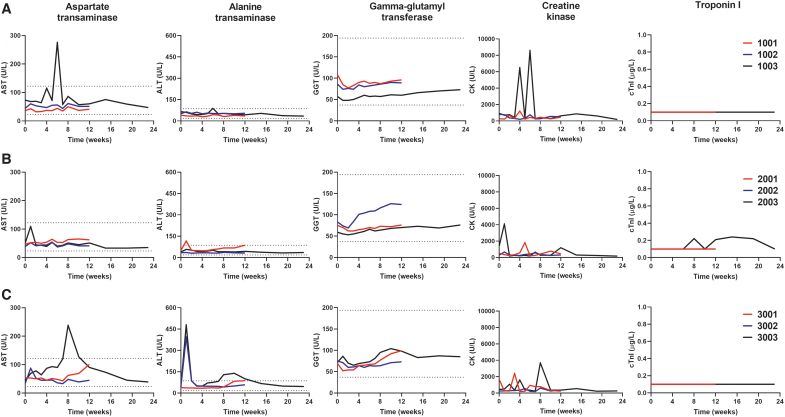
The absence of blood chemistry abnormalities after systemic vector delivery. NHPs (*n* = 9) were systemically treated with **(A)** diluent: animals 1001, 1002, 1003, or scAAV9.U7.ACCA vector at either **(B)** low dose (3 × 10^13^ vg/kg): animals 2001, 2002, 2003, or **(C)** high dose (8 × 10^13^ vg/kg): animals 3001, 3002, 3003. Blood samples were collected before and after vector administration for up to 12 weeks (3-month time point, *red and blue lines*) or 23 weeks (6-month time point, *black lines*). Data shown as absolute numbers per liter in all animals. *Dashed lines* indicate the reference values provided by Charles River laboratory. ALT, serum alanine aminotransferase; AST, serum aspartate aminotransferase; CK, serum creatine kinase; cTnI, cardiac troponin I; GGT, serum gamma glutamyl transferase; NHP, nonhuman primate.

Laboratory assessments included clinical chemistry, troponin I, and cardiac biomarkers' hematology, coagulation, urinalysis, and bone marrow cytology. Individual animal results for all assessments are not presented, but key serum clinical chemistry values are summarized in Fig. 1. The serum aspartate transaminase (AST) stayed within the normal range expected for juvenile cynomolgus throughout observation, with the exception of transient elevations observed in both the diluent-treated animal at 6 weeks postinjection (increasing to 3.8 × the baseline value, and 2.3 × times the upper limit of normal [ULN]), and in one high-dose-treated animal at 8 weeks postinjection (to 5.4 × baseline, and 2.0 × times ULN). Serum alanine transaminase (ALT), however, demonstrated a transient elevation by day 7 postinjection in three of the six treated animals. At the low dose, one animal had a minimal elevation (2.4 × baseline, but only 1.3 × ULN), whereas two of the high-dose-treated animals showed larger elevations (animal 3002, 9.2 × baseline, 4.6 × ULN; animal 3003, 11.1 × baseline, 5.6 × ULN). All of these elevations returned to normal range by 2 weeks postinjection. Only one animal in the high-dose group (3003) showed mild transient elevation in ALT later at the 8-, 10-, and 12-week time points. The values maximized at 10 weeks at 3.2 × baseline and 1.6 × ULN before returning to normal range, and were not associated with any clinical findings. The serum gamma-glutamyl transferase showed baseline variability in either diluent- or vector-treated NHPs with minor elevations (not exceeding the ULN) that could be detected in all NHP groups, leading to the conclusion that those variations are not scAAV9.U7.ACCA-related.

Serum creatine kinase (CK) levels for individual animals are summarized in Fig. 1. These varied during the observation period, which may occur with routine handling of animals under study conditions. The highest elevations were seen in diluent-treated animal 1003, in which transient elevations were noted at the 4-week (to 7.1 × baseline) and 6-week (9.4 × baseline) time points, returning to a value lower than baseline at the intervening 5-week time point. The high-dose-treated animal 3003 also had an elevation in CK at the 8-week time point (8.3 × baseline). We note that the CK elevations at these time points coincided with elevations in AST in these animals (Fig. 1), consistent with transaminase release from muscle as seen in clinical observations^15^ and suggesting that the AST elevations may not be related to release from the liver.

Cardiac troponin I (cTnI) (Fig. 1) was obtained at baseline and after administration of vector. Eight of nine animals maintained cTnI levels below the limits of quantification (0.1 μg/L). Only animal 2003, treated at the low dose (3 × 10^13^ vg/kg), showed a minimal cTnI increase (2.4 × ) between 8 and 19 weeks postinjection, the level of which returned to baseline in week 23; such variability in cTnI levels in NHPs has been previously described.^16–18^ The cardiac biomarker CK-MB showed no significant difference among animals at either 3 or 6 months (as did neither of the other isoforms, CK-MM and CK-BB). Urinalysis parameters remained normal throughout the study.

**Figure 2.**
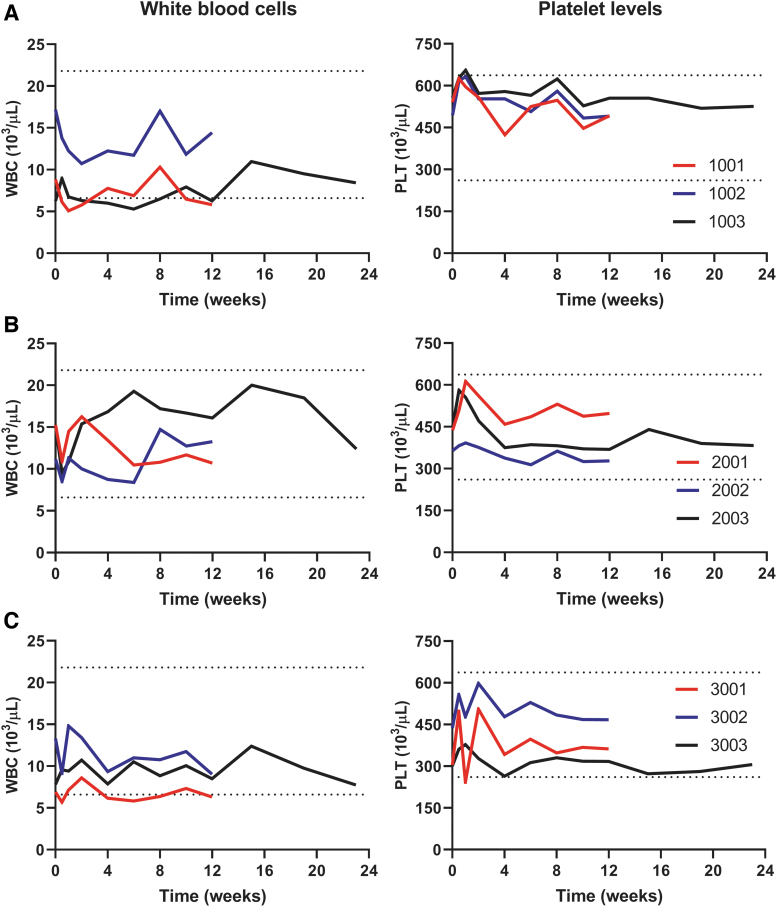
The absence of hematologic abnormalities after systemic vector delivery. NHPs (*n* = 9) were systemically treated with **(A)** diluent: animals 1001, 1002, 1003, or scAAV9.U7.ACCA vector at either **(B)** low dose (3 × 10^13^ vg/kg): animals 2001, 2002, 2003, or **(C)** high dose (8 × 10^13^ vg/kg): animals 3001, 3002, 3003. Blood samples were collected before and after vector administration for up to 12 weeks (3-month time point, *red and blue lines*) or 23 weeks (6-month time point, *black lines*). Data shown as absolute numbers per microliter in all animals. *Dashed lines* indicate the reference values provided by Charles River laboratory. PLT, platelet count; WBC, white blood cells.

Hematology values are summarized in Fig. 2. No specific changes in white blood cells or platelet levels were noted, and other parameters remained normal in all animals. Minimal monocyte increase (1.4 × to 4.5 × ) and large unstained cell elevation (1.5 × to 5.3 × ) were observed at both doses in the period of 1–2 weeks postvector administration, which were likely related to increased monocyte counts, with values that were generally comparable with diluent control and/or baseline by week 4. Coagulation parameters showed a slight decrease of fibrinogen (0.6 × to 0.8 × ) for one animal (3003) at the high dose of scAAV9.U7.ACCA (8 × 10^13^ vg/kg) in week 2 through 12, the level of which was returned to baseline value approximately by week 15.

Postmortem bone marrow cytology tests displayed no changes in the ratio of myeloid to erythrocyte precursors (M:E ratio) at either dose level at the time of testing (weeks 13 and 26). In all animals, the hematopoietic lineages (erythroid, myeloid, and megakaryocytic) were observed in adequate maturation sequences and exhibited normal morphology. Two animals—2002 at the low dose (3 × 10^13^ vg/kg) and 3001 at the high dose (8 × 10^13^ vg/kg)—had mildly increased lymphocyte percentages at time of test performance, week 13. At 6 months postvector administration, all animals showed no significant changes in lymphocyte percentages.

The T cell response to the vector capsid was evaluated by interferon gamma ELISpot assay. No positive response to AAV9 capsid was detected at any of the time points tested for all nine animals. Some of the animals showed reactivity to cynomolgus CMV (peptide antigen) and all of them were responsive to ConA (mitogen) used as a positive control.

### Gross pathologic and histopathologic assessments

All nine animals tolerated the treatment with either diluent or scAAV9.U7.ACCA vector at both doses well with no toxicity observed. All animals were necropsied as scheduled. No gross necropsy findings were found in any of the nine animals treated with either diluent or scAAV9.U7.ACCA vector at either dose. At necropsy, there were no significant body or organ weight changes attributable to administration of scAAV9.U7.ACCA.

At 3 months postinjection, minimal single-cell hepatocyte necrosis with or without associated mixed cell infiltrates was observed in one animal (2001) at the low dose and both animals (3001 and 3002) at the high dose. In addition, mild diffuse hepatocellular vacuolization was observed in animal 3001 at 8 × 10^13^ vg/kg dose. Vacuolated hepatocytes were rounded with large cytoplasmic vacuoles that lacked well-defined margins. Minimal mixed inflammation and acute hemorrhage (with fibrin exudation) were noted in the synovium lining the stifle joint (distal femur) of one 8 × 10^13^ vg/kg dose group male (animal 3002). Due to the isolated incident and acute nature of histological changes, this finding was considered to be incidental and unrelated to scAAV9.U7.ACCA administration.

At 6 months postinjection, the minimal nonspecific histopathologic findings noted in the liver of animals euthanized at 3 months postinjection were not observed. All other microscopic findings observed were considered procedural-related, incidental, of the nature commonly observed in male juvenile cynomolgus monkeys,^19–22^ and/or were of similar incidence and severity in control and dosed animals and, therefore, were considered unrelated to the administration of scAAV9.U7.ACCA.

### Biodistribution

**Figure 3.**
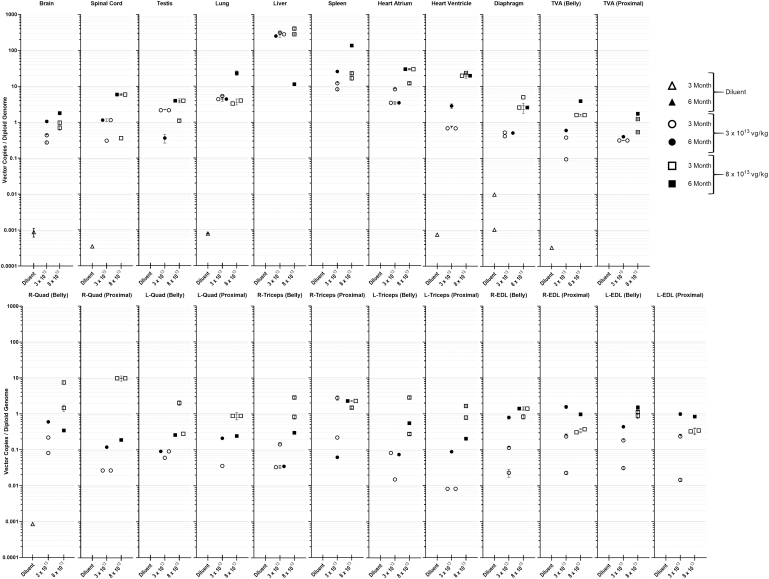
Biodistribution of scAAV9.U7.ACCA vector copies (vc) per diploid genome (dg) in a range of tissues. *Macaca fascicularis* (*n* = 9) were systemically injected with either diluent (*open and filled triangle*) or scAAV9.U7.ACCA vector at 3 × 10^13^ vg/kg (*open and filled circles*) and 8 × 10^13^ vg/kg (*open and filled squares*) doses. Symbols represent animals euthanized at either 3 months (*open*) or 6 months (*filled*) postinjection. scAAV9.U7.ACCA vector was not detectable below 0.0006 vc/dg in NHPs dosed with diluent (control) and is not displayed in the graph. The 23 tissues analyzed from all animals were run in technical triplicates and reported as mean ± SD represented on the logarithmic grouped scatter plot above whenever possible. SD, standard deviation.

Biodistribution of scAAV9.U7.ACCA transgene was assessed by quantitative real-time PCR (qPCR) using DNA extracted from major organs (heart, liver, lung, spleen, testes, brain, and spinal cord); selected skeletal muscles (transverse abdominis, and, bilaterally, the quadriceps femoris (Quad), triceps brachii (Tri), and extensor digitorum longus (EDL); diaphragm; and two areas within the heart (left atrium and ventricle). Within each skeletal muscle, a sample was obtained both from the central (belly) portion of the muscle and from a more proximal section for independent analysis. The detailed results are presented in Fig. 3.

As expected, the highest values for vector copies per diploid genome (vc/dg) were found in the liver and spleen. At 3 months postinjection, liver contained (rounded to two decimals) 252.64 or 315.59 (mean, 284.12) vc/dg in the 3 × 10^13^ vg/kg (low dose) animals, and 284.33 or 406.49 (mean, 354.41) vc/dg in the 8 × 10^13^ vg/kg (high dose) animals. In the 6-month animals, liver contained 281.78 vg/dg (low dose) and 11.48 vc/dg (high dose). The spleens at 3 months contained 8.24 or 12.04 (mean, 10.14) vc/dg (low dose) and 16.72 or 22.93 (mean, 19.82) vc/dg (high dose). At 6 months, the spleen contained 25.73 vc/dg (low dose) and 135.24 vc/dg (high dose). Despite the high number of vector copies in the tissue, these were not associated with toxicity or inflammation in hepatocytes or spleen cells.

Among skeletal muscles, values at 3 months in the low-dose animals ranged (in vc/dg) from 0.01 to 2.78, with both values found in triceps samples. At 3 months in the high-dose animals, values ranged from 0.08 (Tri) to 09.86 (Quad). In the 6-month animals, the values at the low dose ranged from 0.40 (Tri and TVA) to 1.58 (EDL); at the high dose, they ranged from 0.19 (Quad) to 3.93 (TVA). In the heart, values in (vc/dg) at 3 months ranged from 0.68 (ventricle) to 8.27 (atrium) at the low dose, and from 12.10 (atrium) to 23.50 (ventricle) at the high dose. At 6 months, the values at the low dose ranged from 2.85 to 19.73 (ventricle) or from 3.45 to 29.90 (atrium). In the diaphragm (Dia), at 3 months, values ranged from 0.41 to 0.53 (low dose) and from 2.05 to 5.03 (high dose); at 6 months, the values were 0.51 (low dose) and 2.61 (high dose).

### Quantification of exon 2 skipping by reverse transcription polymerase chain reaction

We have previously shown that the systemic delivery of scAAV9.U7.ACCA vector in Dup2 mice results in robust skipping of exon 2, which leads to an mRNA that contains either a single exon 2 (WT mRNA) or no copies of exon 2 (Del2 mRNA); unskipped transcript contains the duplicated exon 2.^14^ Both mRNA transcripts are therapeutic, because the Del2 transcript is translated via utilization of the exon 5-localized IRES, resulting in an N-terminal deleted yet highly functional dystrophin, with translation beginning at an AUG codon at exon6.^14^ Because cynomolgus monkeys contain a WT *DMD* locus, all animals treated with the vector would be expected to demonstrate only WT transcript (signaling no exon 2 skipping) or the Del2 transcript.

**Figure 4.**
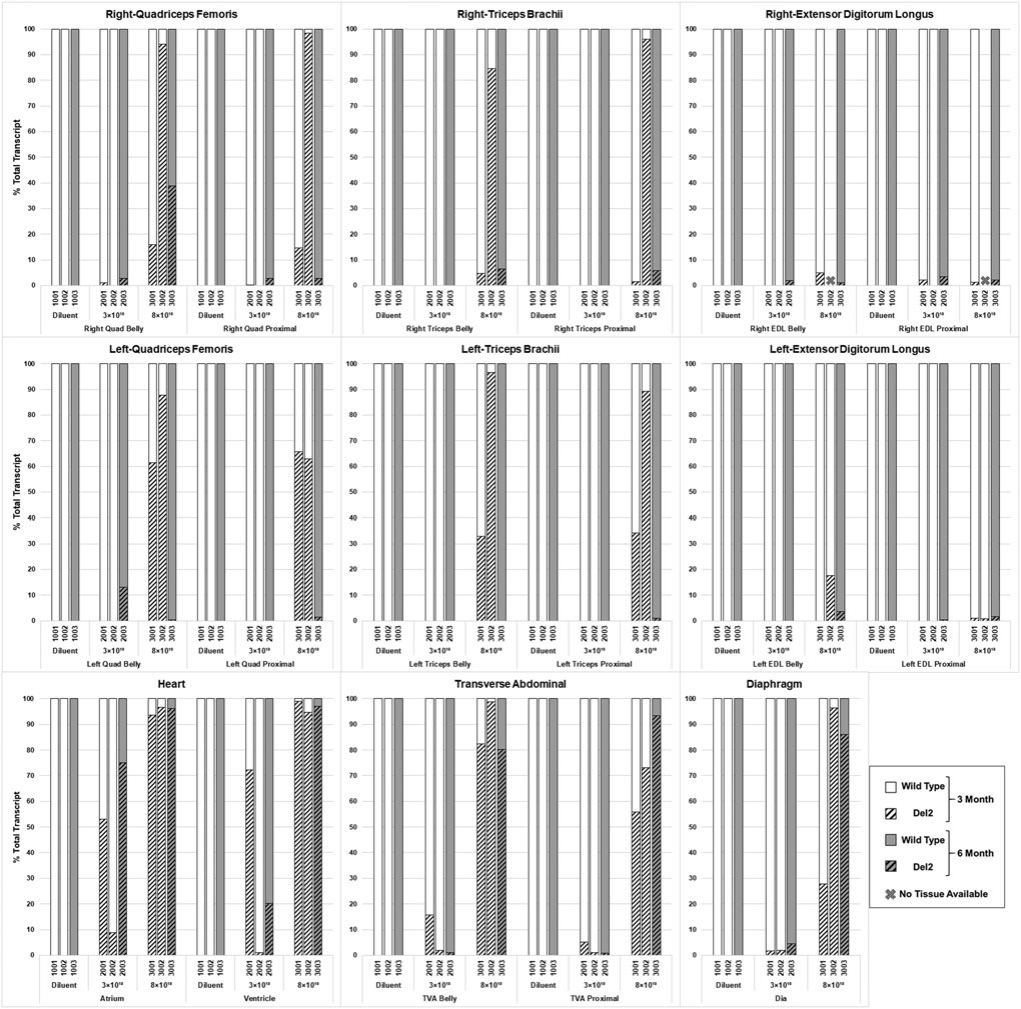
Quantification of exon 2 skipping by RT-PCR, represented as percentage of total *DMD* transcript. *Macaca fascicularis* (*n* = 9) were systemically treated with diluent (control) or low (3 × 10^13^ vg/kg) and high (8 × 10^13^ vg/kg) doses of scAAV9.U7.ACCA. Six of the nine animals were sacrificed at 3 months (*n* = 2, *white background*) and three at 6 months (*n* = 1, *gray background*) postinjection. Eleven unique muscle areas from each animal were evaluated (bilaterally when possible) by RT-PCR, as labeled under each group. “Proximal” and “Belly” are described in Materials and Methods. Animal numbers are as described in Fig. 1 legend. Each tissue sample was run as a single technical sample and subject to predetermined acceptation criteria. (Two samples—animal 3002, R-EDL Belly and EDL-Proximal—had insufficient tissue for RNA extraction, and marked with a symbol “X”). DMD, Duchenne muscular dystrophy; EDL, extensor digitorum longus; RT-PCR, reverse transcription polymerase chain reaction.

Quantification of exon 2 skipping by reverse transcription polymerase chain reaction (RT-PCR) is summarized in Fig. 4. All skeletal muscles were assessed at two different regions to evaluate variation of skipping in one muscle. The regions are defined as “proximal” (a sample from the region closer to the proximal myotendinous junction) and “belly” (a sample near the midsection of the muscle). Images of all agarose gels are represented in Supplementary Figs. S1 and S2. At 3 months postinjection, a dose response in exon skipping was observed. In the two animals treated with a dose of 8 × 10^13^ vg/kg (animal 3001 and 3002), robust exon 2 skipping was seen in nearly all muscles evaluated by RT-PCR. This ranged from 14.3% to 98.8% in the Quad; from 0.8% to 96.7% in the Tri; and from 55.9% to 98.8% in the TVA. A lesser degree of skipping was seen in the EDL, ranging from 0% to 17.7%. Skipping was particularly robust in the heart, with levels of 93.7% and 96.6% in the atrium, and 94.8% and 99.1% in the ventricle. Skipping in the Dia was seen at 27.9% and 96.4%. Greater variation was seen in the two animals treated with the lower dose of 3 × 10^13^ vg/kg, particularly in the heart. In the atria, animal 2001 had 53.1% skipping, whereas animal 2002 had 8.9% skipping; in the ventricles, animal 2001 had 72.3%, whereas animal 2002 had 1.1% skipping. Skeletal muscle skipping was less pronounced at this lower dose, with 0% to 0.7% in the Quad, 0% to 2.1% in the EDL, 0% to 0.1% in the Tri, and with 1.1% to 15.7% in the TVA; skipping in the Dia ranged from 1.7% to 1.9%.

One animal in each group was maintained until 6 months postinjection. A dose of 8 × 10^13^ vg/kg (animal 3003) showed maintenance of high levels of skipping in the heart (atrium, 96.3%; ventricle, 97.0%) that was observed in 3-month animals and skipping in the Dia of 86.1%. Skipping in skeletal muscle ranged in Quad from 0.3% to 38.9%; in Tri, 0–6.6%; in TVA, 80.3–93.5%; and in EDL, 1.0–3.7%. A dose of 3 × 10^13^ vg/kg dose (animal 2003) resulted in skipping of 75.1% in the atria, 20.3% in the ventricle, and 4.5% in the Dia. Skipping in skeletal muscle ranged in Quad from 0% to 13.1%; in Tri, 0% (no skipping); in TVA, 0.9–1.1%; and in EDL, 0–3.4%. As expected, no exon 2 skipping was observed in the control animals at 3 (animals 1001 and 1002) or 6 (animal 1003) months.

### RNA sequencing analysis in skeletal muscle

As a second test of biological effect, analysis of the *DMD* mRNA was performed in the heart, diaphragm, and quadriceps using RNA sequencing (RNA-seq). Each sample was sequenced to between 100 and 230 million reads (3-month phase animals) and 135 and 165 million reads (6-month phase animals) to ensure adequate coverage of *DMD* for exon skipping analysis. The sample number in each treatment group (*n* = 1 or 2) precluded statistical significance testing in comparing median *DMD* transcript or exon junction counts between groups.

**Figure 5.**
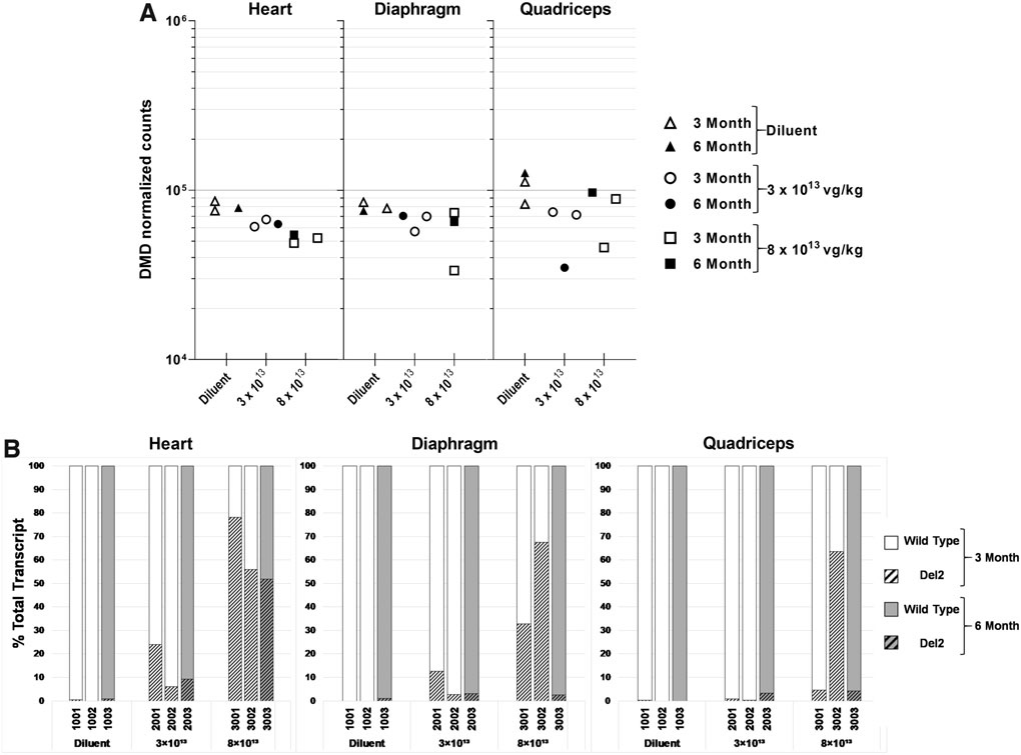
Analysis of exon 2 exclusion by RNA-Seq. **(A)** Endogenous DMD mRNA transcript counts (all transcripts) in the heart, diaphragm, and quadriceps samples, 3 (*open symbols*) or 6 (*filled symbols*) months of delivery of diluent (*triangles*), 3 × 10^13^ vg/kg (*circles*), or 8 × 10^13^ vg/kg (*squares*) of scAAV9.U7.ACCA vector. **(B)** Percentage of DMD exon 2 skipping by tissue. WT and Del2 transcripts are displayed as a *solid upper portion* and *hashed bottom portion* of bar, respectively. Data shown as individual animal data. Del2, deletion exon 2; RNA-Seq, RNA sequencing; WT, wild type.

As measured in the 3-month animals, the RNA level of DMD decreased in a dose-dependent manner in all analyzed tissues in postvector administration compared with the control group. Animals demonstrated a 21–25% and 31–38% reduction in *DMD* mRNA level at low and high doses, respectively (Fig. 5A and Supplementary Table S1). In the 6-month animals, a dose-dependent decrease of total *DMD* mRNA level was observed in the scAAV9.U7.ACCA-treated heart and diaphragm tissues. In these two tissues, animals treated with either low or high doses demonstrated a 7–20% and 14–31% reduction in *DMD* mRNA level, respectively. In the quadriceps, the animal treated with the higher dose exhibits a 23% reduction in *DMD* mRNA, which is comparable with the reduction in the heart and diaphragm, whereas in the quadriceps, the lower dose was associated with a 72% reduction in *DMD* mRNA (Fig. 5A and Supplementary Table S1).

Skipping of *DMD* exon 2 was quantified as the percentage of reads spanning exon junction 1–3 in reads spanning exon junctions 1–2, 2–3, and 1–3. At 3 months, a dose-dependent skipping of exon 2 was observed in all three tested tissues at both low and high doses, although it was more pronounced at the higher dose, with means of 34% (Quad), 50% (Dia), and 67% (Heart) (Fig. 5B and Supplementary Table S2). At 6 months, the diluent-treated tissue samples showed little to no exon 2 skipping. Dose-dependent skipping was most pronounced in the heart, with 9% at the lower and 52% at the higher dose. In contrast, the Quad and Dia exhibited low levels (3–4%) of exon 2 skipping at 6 months (Fig. 5B and Supplementary Table S2). There was general concordance between quantifications between the RT-PCR and RNA-seq results, and although we note the discrepant results seen in the diaphragm of animal 3003, we can postulate several potential reasons for this sole discrepancy. We note the differences in methodology (RT-PCR vs. RNA-Seq), note that the RNA extractions were done in different laboratories using different techniques, and finally note that RT-PCR could potentially overestimate the amount of Del2 transcript due to preferential amplification of shorter transcripts.

## Discussion

The use of U7snRNA-mediated therapy for targeted exon skipping to restore an open reading frame in patients with out-of-frame mutations has demonstrated significant promise in animal models of severe hereditary diseases such as β-thalassemia,^2,23^ spinal muscular atrophy,^5,6,24^ and DMD.^14,25–28^ In the particular setting of duplications of single exons within the *DMD* gene, such a therapy would likely be preferential to microdystrophin therapies, despite their significant promise,^29–37^ as exclusion of one of the two copies of a duplicated exon will result in expression of a full-length, WT dystrophin protein rather than an engineered microdystrophin protein.

Here we have demonstrated, for the first time in primates, the absence of toxicity of such a U7snRNA vector, providing evidence for the safety of this approach in humans. The main purpose of this study was to evaluate the safety of systemic delivery of the scAAV9.U7.ACCA to NHPs at clinically relevant doses, beginning at the anticipated minimal efficacious dose (as determined in studies of systemic delivery in the Dup2 mouse model) of 3 × 10^13^ vg/kg and a higher dose of 8 × 10^13^ vg/kg, doses planned for the current clinical trial of gene transfer in Dup2 patients (NCT04240314). The results establish the safety of a single intravenous infusion at these doses, with no adverse effects or detectable toxicity in all animals during either 3 or 6 months of observation, as judged by clinical, biochemical, or histopathologic measures.

A critical concern for clinical gene therapy is the degree of immune responses to the viral capsid and/or transgene product. Importantly, we saw no evidence for immune-mediated toxicity, and specifically, we did not see any evidence of abnormalities in the dorsal root ganglia similar to those described with use of an AAV9 vector at a slightly higher dose.^38^ Indeed, we have not detected ELISpot responses to rAAV9 capsid in any of the vector-treated animals at any of the time points, suggesting that T cells were not adequately primed by intravenous delivered rAAV9 vector, or most likely they existed at levels below the threshold of detection, and this was correlated with the absence of detectable toxicity and/or tissue damage in the treated NHPs after long-term assessment. Antibody and T cell responses to the transgenes were not evaluated, as U7snRNAs are noncoding and no protein is expressed.

The biodistribution of the scAAV9.U7.ACCA was consistent with expectations for AAV9 vectors, for which efficient transduction of both skeletal and cardiac muscles, the central nervous system, and a wide variety of organs such as the liver and lung at high efficiency following systemic vector administration has been described.^39–45^ Although the highest levels of transduction efficiency were seen as expected in the liver and spleen, significant and generally dose-dependent transduction was observed in skeletal muscles, with particular efficiency in transduction of the heart and diaphragm.

Consistent with the anticipated mechanism of action of the vector, we observed exclusion of exon 2 from the *DMD* mRNA. This appeared more robust using an RT-PCR assay than via RNA-Seq; this lack of concordance remains unexplained. Nevertheless, skipping appeared to occur in a generally dose-dependent manner, particularly in the heart. Such robust skipping in the heart, likely related, in part, to tropism of AAV9 for that organ, will be of clinical relevance in treating boys with DMD, as cardiomyopathy is a significant cause of morbidity and mortality.^46^ We have not tried to assign a therapeutic degree of skipping in this WT *DMD* context, as unpublished data from the preclinical development program demonstrate a higher degree of exon 2 skipping in the Dup2 context in comparison with the WT context. We note that the quantification of exon skipping by either the RT-PCR or RNA-seq methods reflects only the steady-state levels of transcripts, and due to presumed or potential differences in stability of the different transcripts, may not precisely reflect the actual rate of skipping overall. Nevertheless, “percentage skipping” is commonly used as used here, and allows some general comparison between methods or doses. Based on the presence of the IRES within exon 5 that is utilized in the absence of exon 2, we predicted that complete skipping would result in no significant muscle pathology, and histopathological examination confirmed the absence of visible lesions in all skeletal muscles. We acknowledge that there is the potential that sequence-specific side effects may not be adequately addressed in animal models, and that off-target splicing variation should be assessed in future clinical trials.

Overall, our results demonstrate that intravenous delivery of scAAV9.U7.ACCA in male juvenile cynomolgus monkeys at 3 × 10^13^ vg/kg or at 8 × 10^13^ vg/kg was safe and well tolerated, with no evidence of toxicity, providing strong evidence to support the initiation of a first-in-human trial of U7sRNA therapy. More generally, they provide the first rigorous demonstration of the safety of systemic use of this therapeutic approach in primates. They suggest that therapeutic expression of exogenous U7snRNAs does not result in significant observable pathology, and argue for the continued development of this therapeutic approach for human heritable diseases.

## Materials and Methods

The entire study was performed at a contract research organization (Charles River Laboratories, Reno, NV) in accordance with the principles of good laboratory practice (GLP), with the exception of the RT-PCR analysis of exon skipping and qPCR analysis of biodistribution (performed in the Flanigan laboratory), the interferon-γ enzyme-linked immunosorbent spot (ELISpot) assay (performed at the NCH Research Immune Monitoring Core), and anti-AAV9 ELISAs (performed at the University of Pennsylvania Immunology Core).

### scAAV9.U7.ACCA vector production, preparation, and delivery

The scAAV9.U7.ACCA viral vector^14^ was produced by the Clinical Manufacturing Facility at Nationwide Children's Hospital under the principles of cGMP for use in a Phase I/IIa clinical trial. Physical titer determination was based on degradation of nonencapsidated DNA following digestion of viral capsids. Released encapsidated DNA was quantified by quantitative real-time PCR (qPCR) to determine the DNase Resistant Particle (DRP) titer, utilizing a unique probe directed toward the U7 promoter and a linearized plasmid standard, and stored in a −80°C freezer until use. On day of dosing, the test article dosing formulation was prepared by dilution with control article at an appropriate concentration to meet dose-level requirements. The vector was delivered in the diluent TMN200 (containing 20 mM Tris pH 8.0, 1.0 mM MgCl_2_, 200 mM NaCl, 0.001% Pluronic 188) that was manufactured at Audentes Therapeutics following GLP regulations. For control animals (Group 1), TMN200 was diluted with Lactated Ringer Solution (LRS) (90:10 v/v TMN200:LRS) before administration. For each animal, the test article (diluent or vector) was delivered to either the right or left cephalic vein over 30 min in a volume of 8 mL/kg.

### Animals

Nine male cynomolgus monkeys (*Macaca fascicularis*) ranging in age from 2.5 to 2.7 years were housed and cared for at Charles River Laboratories in accordance with the Guide for the Care and Use of Laboratory Animals.^47^ All animal procedures were approved by Charles River Laboratories' Animal Use Committee, and the study was conducted in compliance with principles of GLP. Animals were assigned to groups (Table 1) based on the results from the prescreening for AAV9 neutralizing antibody (NAb) titer, with dilution less than <1:5. Substitutions were not made after initial group assignment. Additional criteria for selection included at least acceptable results from the pretreatment echocardiograms and ophthalmic examinations. Details on conditions of housing are provided in the Supplementary Data.

### Biochemical, hematologic, and immunologic studies

Animals were fasted before clinical chemistry blood collections. Blood for ELISpot analysis was collected from each animal 3 days before treatment, week 5 and 13 (all study animals), and week 26 (animals 1003, 2003, and 3003). Samples were shipped to IQ Biosciences for isolation of PBMCs before shipment to Nationwide Children's Hospital for ELISpot analysis.

### Sample collection and analysis

Necropsy was conducted at the Charles River laboratory in accordance with GLP regulations. Animals were fasted overnight before their scheduled necropsy. Animals in each dose group were euthanized and necropsied at the same necropsy station. Aseptic technique was used to collect tissues to avoid cross contamination between dose groups. Histopathologic samples were preserved in 10% v/v neutral buffered formalin and shipped to a board-certified veterinary pathologist for histopathological evaluation. Other tissues were snap frozen in liquid nitrogen or (for muscle) isopentane cooled to liquid nitrogen temperatures and stored at −80°C until used.

### RT-PCR quantification of exon 2 skipping

Skipping of exon 2 was quantified by RT-PCR of mRNA derived from skeletal muscles, diaphragm, or heart. Total RNA was extracted from frozen tissues using RNA Clean & Concentrator-25 according to the manufacturer's protocol (catalog #R1018; Zymo Research, Tustin, CA). For each sample, 1 μg of total RNA was used to generate cDNA using random hexamer primers according to the manufacturer's protocol (catalog #K1691, RevertAid RT Kit; Thermo Scientific, Waltham, MA). cDNA was amplified via PCR (catalog #K0171; Thermo Fisher, Waltham, MA) using primers specific to the DMD 5′UTR and the exon 3–4 junction (sequences available upon request; IDT, Coralville, IA). Digital images of DNA bands on gels stained with ethidium bromide as an intercalating agent were quantified using ImageJ software (Version 1.46r; NIH) to determine relative amounts of different amplicons.

### Quantitative PCR

Vector genomes were quantified in skeletal muscles (quadriceps, triceps, TVA, EDL), heart, diaphragm, spleen, liver, testis, brain, spinal cord, and lung. Total DNA was extracted from tissue samples using the DNeasy Blood & Tissue Kit (catalog #69506; Qiagen, Germantown, MD), and vector genomes quantified by quantitative PCR (qPCR) using a linearized AAV.U7.ACCA plasmid to generate a standard curve allowing absolute quantification. The DNA samples were analyzed using TaqMan Universal PCR Master Mix (catalog #4304437; Applied Biosystems, Foster City, CA) and 7500 Real-Time PCR System (Applied Biosystems), following the procedures recommended by the manufacturer and a set of primers unique to the transgene (sequences available upon request). Genomic DNA from diluent-injected NHP tissues served as experimental control samples. Each sample was run in triplicate. Acceptance criteria for an assay included a standard curve with an R^2^ of 0.9900 or greater, a slope of between −3.0 and −3.8, a Ct value of 18–20 for the point representing 5 × 10^6^ copies per reaction, and undetermined values in the nontemplate control reaction. Further details are included in the Supplementary Data.

### RNA-seq analysis

RNA-seq was performed on quadriceps femoris (Quad), heart, and diaphragm (Dia) for all animals. The frozen tissue samples were shipped to Genewiz, LLC (South Plainfield, NJ) for RNA extraction, library preparation, and sequencing, the details of which are provided in the Supplementary Data. Quality control and analysis of the RNA-seq data were performed using an internally developed bioinformatic pipeline named Bold_Workflow_RNAseq_Star version 2.2.1 (Audentes Therapeutics, Inc.). Details of the analysis are included in the Supplementary Data.

## Supplementary Material

Supplemental data

Supplemental data

Supplemental data

Supplemental data

Supplemental data
