# Supplementary material for: Lack of Toxicity in Nonhuman Primates Receiving Clinically Relevant Doses of an AAV9.U7snRNA Vector Designed to Induce DMD Exon 2 Skipping
Source: Hum Gene Ther. 2021 Sep 23;32(17-18):882–94. doi: 10.1089/hum.2020.286 (PMC10112461; doi:10.1089/hum.2020.286)
Supplement: Supplemental data [file Supp_FigureS1.docx]

**Supplementary Figure S1. Exon 2 skipping in muscle as detected by RT-PCR at 3 months post injection.** Image quantification is described in the text, with results as summarized in Figure 4. Control reactions were run for each group of reactions marked by black border. “N/T” marks samples for which insufficient tissue was available for testing, as discussed in text.

**Supplementary Figure S2. Exon 2 skipping in muscle as detected by RT-PCR at 6 months post injection.** Image quantification is described in the text, with results as summarized in Figure 4. Control reactions were run for each group of reactions marked by black border.
