# Supplementary material for: Lack of Toxicity in Nonhuman Primates Receiving Clinically Relevant Doses of an AAV9.U7snRNA Vector Designed to Induce DMD Exon 2 Skipping
Source: Hum Gene Ther. 2021 Sep 23;32(17-18):882–94. doi: 10.1089/hum.2020.286 (PMC10112461; doi:10.1089/hum.2020.286)
Supplement: Supplemental data [file Supp_TableS1.pdf]

Supplementary Table S1. Normalized endogenous *DMD* counts in diaphragm, heart and quadriceps femoris tissues from non-human primates treated with scAAV9.U7.ACCA.

Tissue	Dose (vg/kg)	Animal ID	DMD normalized counts
**Diaphragm**	Diluent	1001	78333.60
		1002	84981.80
		1003	75735.10
	3×10^{13}	2001	70102.20
		2002	57099.60
		2003	70555.20
	8×10^{13}	3001	73642.60
		3002	33567.40
		3003	65073.20
**Heart**	Diluent	1001	86178.90
		1002	75694.20
		1003	78670.70
	3×10^{13}	2001	60874.50
		2002	67171.80
		2003	63068.50
	8×10^{13}	3001	52156.50
		3002	48698.80
		3003	54612.40
**Quadriceps**	Diluent	1001	112162.80
		1002	82861.30
		1003	126525.20
	3×10^{13}	2001	71693.70
		2002	74262.20
		2003	34906.20
	8×10^{13}	3001	88933.40
		3002	45975.30
		3003	96883.20
