# Supplementary material for: Lack of Toxicity in Nonhuman Primates Receiving Clinically Relevant Doses of an AAV9.U7snRNA Vector Designed to Induce DMD Exon 2 Skipping
Source: Hum Gene Ther. 2021 Sep 23;32(17-18):882–94. doi: 10.1089/hum.2020.286 (PMC10112461; doi:10.1089/hum.2020.286)
Supplement: Supplemental data [file Supp_TableS2.pdf]

Supplementary Table S2. Normalized counts of reads spanning relevant exon junctions in endogenous DMD and percentage of exon 2 skipping and wild-type (WT) transcripts as estimated from those junction counts.

Tissue	Dose (vg/kg)	Animal ID	Exon 1-2	Exon 2-3	Exon 1-3	% Skip	% WT
**Diaphragm**	**Diluent**	1001	1361.6	1556.5	0.0	0.0	100
		1002	1852.6	1767.8	0.0	0.0	100
		1003	1371.1	1417.9	15.3	1.1	98.9
	**3×10^{13}**	2001	1138.1	1161.1	166.6	12.7	87.3
		2002	1264.2	1137.7	33.9	2.7	97.3
		2003	1504.1	1623.3	49.6	3.1	96.9
	**8×10^{13}**	3001	872.9	934.7	441.3	32.8	67.2
		3002	180.2	140.4	333.5	67.5	32.5
		3003	1107.7	1233.8	31.0	2.6	97.4
**Heart**	**Diluent**	1001	1728.9	1685.4	9.2	0.5	99.5
		1002	1286.6	1261.7	0.0	0.0	100
		1003	1473.8	1522.7	13.0	0.9	99.1
	**3×10^{13}**	2001	830.8	770.3	252.2	24.0	76.0
		2002	1068.0	928.4	65.0	6.1	93.9
		2003	1113.5	1071.3	110.2	9.2	90.8
	**8×10^{13}**	3001	179.2	193.5	663.0	78.1	21.9
		3002	231.1	327.6	355.8	56.0	44.0
		3003	426.7	461.3	479.8	51.9	48.1
**Quadriceps**	**Diluent**	1001	2560.4	2318.8	5.1	0.2	99.8
		1002	1557.8	1457.4	0.0	0.0	100
		1003	2352.3	2665.5	0.0	0.0	100
	**3×10^{13}**	2001	1855.8	1635.4	14.0	0.8	99.2
		2002	1677.1	1533.4	4.3	0.3	99.7
		2003	622.4	669.6	21.5	3.2	96.8
	**8×10^{13}**	3001	1636.6	1452.5	72.3	4.5	95.5
		3002	249.7	334.1	509.7	63.6	36.4
		3003	2264.4	2212.4	98.1	4.2	95.8
