# Supplementary material for: Lack of Toxicity in Nonhuman Primates Receiving Clinically Relevant Doses of an AAV9.U7snRNA Vector Designed to Induce DMD Exon 2 Skipping
Source: Hum Gene Ther. 2021 Sep 23;32(17-18):882–94. doi: 10.1089/hum.2020.286 (PMC10112461; doi:10.1089/hum.2020.286)
Supplement: Supplemental data [file Supp_Data.docx]

**Supplemental Materials and Methods**

**Animal Care**

Each cynomolgus monkey was identified using a cage label, animal and tattoo number, and sex. The animals were acclimated to laboratory housing and to chair restraint before the initiation of dosing. Animals were socially-housed (up to 3 animals of same sex and same dosing group together) in stainless-steel cages until 3 months; after completion of the 3-month phase animals were singly housed. Temperatures of 18 °C to 29 °C with a relative humidity of 30% to 70% were maintained. A 12-hour light/12-hour dark cycle was maintained, except when interrupted for designated procedures. Ten or greater air changes per hour with 100% fresh air (no air recirculation) were maintained in the animal rooms.

PMI Nutrition International Certified Primate Chow No. 5048 was provided daily in amounts appropriate for the size and age of the animals. The feed was analyzed by the supplier for nutritional components and environmental contaminants. The diet was supplemented with fruit or vegetables at least 2 to 3 times weekly. Also, small bits of fruit, cereal, or other treats may have been occasionally given to the animals as part of the Testing Facility environmental enrichment program.

Municipal tap water (Reno, NV) after treatment by reverse osmosis and ultraviolet (UV) irradiation was freely available to each animal via an automatic watering system (except during designated procedures). Periodic analysis of the water was performed. There were no contaminants known to be present in the water at levels that would interfere with the results of this study.

Animals were socialized to provide for psychological enrichment, with the exception of times when they were separated for designated study procedures/activities. Animals were provided with additional psychological enrichments (e.g., Aquasol paper, mung bean, or cellophane noodles, etc.) at least twice per week. Up to 3 animals of the same sex were commingled within dose groups after initial compatibility tests were completed except after end of 3-month phase animals were singly housed. Animals were separated during designated procedures/activities.

***Immune monitoring***

ELISAs were performed at the University of Pennsylvania Immunology core. All animals were screening for AAV9 neutralizing antibody (NAb) titer prior to transfer to study. T cell immunity to rAAV capsid using a highly sensitive enzyme-linked immunosorbent spot (ELISpot) assay was completed before gene transfer and at time points Day 35, Day 90, and Day 181using a human interferon gamma (IFN-γ) ELISpot kit from Cellular Technology Limited (CTL). Briefly, PBMCs were added to 96-well plates coated with an anti-IFN-γ capture antibody and cultured with one of the following test antigens in duplicate: peptides AAV9 pool [1-60], AAV9 pool 2[61-121], AAV9 pool 3[122-182], CyUL55CMV (positive peptide control), CyUL83CMV (positive peptide control), ConA (positive mitogen control), or DMSO (negative control). A biological reference sample with known reactivity to CMV pp65 was also plated (assay control). Cells were incubated for 24 h period at 37°C in a humidified incubator, 5% CO2. After 24 h, the plates were washed two times with PBS, followed by washing two times with 0.05% Tween-PBS. An anti-human IFN-γ detection solution was then added and allowed to incubate for 2 h at room temperature. Plates were then washed three times with 0.05% Tween-PBS and Tertiary solution was added and allowed to incubate for 30 min at room temperature. Plates were washed two times with 0.05% Tween-PBS and then two times with distilled water. Blue Developer Solution was added and allowed to incubate for 15 min at room temperature to develop chromogen. Plates were gently rinsed three times with tap water and allowed to dry a minimum of 4 h. After drying, the plates were imaged.

***Echocardiograms***

Echocardiograms were performed once pretreatment, and again during the week prior to each necropsy. The animals were restrained (sedated) for each echocardiogram measurement in lateral recumbency on a purpose-designed table, to allow placement of the transducer on the dependent side of the thorax. The animals were shaved (approximately 4”x4” on the left and right axillary region) to facilitate the imaging. All examinations were performed by a board-certified veterinary cardiologist and the echocardiograms were obtained with a GE Vivid i echocardiographic recorder. Two-dimensional, M-mode and Doppler measurements were performed with a 3.5/6.9 MHz phased array sector transducer. All measurements to the extent possible were taken in triplicate to obtain an average.

***RNA-Seq sample preparation***

Total RNA of the frozen tissue samples was extracted using mirVana miRNA Isolation Kit (Thermo Fisher Scientific), and the quality and quantity of RNA were assessed by Nanodrop, Qubit, and TapeStation. RNA-seq libraries were prepared using Illumina TruSeq Stranded RNA Library Prep Kit with Ribo-Zero Gold and TruSeq RNA Unique Dual Indexes for sample multiplexing. ERCC (External RNA Control Consortium) RNA Spike-In Mixes (Thermo Fisher Scientific) were added during the library prep to control for various sources of variability. For each type of tissue, all samples were multiplexed and sequenced together across one lane of an Illumina HiSeq 4000 (2 x 150bp) for a total of three lanes of raw data.

***RNA-Seq analysis***

The workflow is comprised of two parts: part 1 is run for each sample and part 2 combines the data across samples. Both parts are run on the DNAnexus cloud computing platform. For part 1, the paired fastqs for each sample were run through FastQC (Babraham Bioinformatics, version 0.11.3) to obtain general QC metrics. Adapters were trimmed by Skewer^1^ (version 0.2.2), then reads were aligned with STAR^2^ (version 2.5.3a) using a genome index built from the M. fascicularis reference genome (MFas5.0), Ensembl version 91 transcript annotations, and the scAAV9.U7.ACCA transgene coding sequence(s). The alignments in BAM format were run through Picard’s CollectRNASeqMetrics (version 2.18.2) for post-alignment quality control.

In part 2, the output from FastQC and Picard along with the logs from Skewer and STAR from all samples were combined by MultiQC^3^ (version 1.5) for easier review of QC results. In a separate analysis, gene counts from STAR were normalized for sequencing depth by DESeq2^4^ (version 1.18.1). This resulted in normalized read counts for each gene in each sample, including DMD. The normalized read counts per gene represent the RNA level from all transcripts of that gene. Counts for reads spanning DMD splice junctions 1-2, 2-3, and 1-3 were obtained from the STAR sj.out files, and were normalized by dividing by each sample’s size factor obtained from DESeq2. The percentage of exon 2 skipping was then calculated as n1-3/(n1-3 + mean (n1-2, n2-3))*100, where ni-j is the number of reads spanning the junction between exon i and exon j. The percentage of exon 2 inclusion was calculated as mean (n1-2, n2-3)/(n1-3 + mean (n1-2, n2-3))*100.

***Quantification of vector copy number by qPCR***

The absolute quantification of vector genome copies present in a sample was determined by comparison of the cycle, at which the threshold of detection is crossed (Ct value) in respect to the value derived from the standard curve run with each assay. Each standard curve dilution as well as each test sample were run in triplicate for enhanced accuracy. The number of vector copies per diploid genome (vc/dg) is calculated by taking the absolute DNA value derived from the standard curve and dividing by the number of diploid genomes per nanogram of DNA (166.67 genomes/ng)^5^. The samples with fewer than 0.0006 vc/dg are considered to be negative. While samples that have 0.0006 vc/dg or more are considered to be positive. Sample reactions that did not pass the threshold by the end of the 40 cycles were reported as “undetermined” by the system’s software, and thus considered below the lower limit of quantification (BLLOQ). If two of the three replicates in the assay were undetermined, the replicate that did return a specific copy number was reported as the “mean” with no standard deviation. If one of the three replicates was undetermined, the mean and standard deviation is calculated from the two replicates that generated a value. If all three replicates fail to generate a value the mean was reported as undetermined and no standard deviation is provided.

**References**

1 Jiang, H., Lei, R., Ding, S. W. & Zhu, S. Skewer: a fast and accurate adapter trimmer for next-generation sequencing paired-end reads. *BMC Bioinformatics* **15**, 182, doi:10.1186/1471-2105-15-182 (2014).

2 Dobin, A. *et al.* STAR: ultrafast universal RNA-seq aligner. *Bioinformatics* **29**, 15-21, doi:10.1093/bioinformatics/bts635 (2013).

3 Ewels, P., Magnusson, M., Lundin, S. & Kaller, M. MultiQC: summarize analysis results for multiple tools and samples in a single report. *Bioinformatics* **32**, 3047-3048, doi:10.1093/bioinformatics/btw354 (2016).

4 Love, M. I., Huber, W. & Anders, S. Moderated estimation of fold change and dispersion for RNA-seq data with DESeq2. *Genome Biol* **15**, 550, doi:10.1186/s13059-014-0550-8 (2014).

5 Berry, G. E. & Tse, L. V. Virus Binding and Internalization Assay for Adeno-associated Virus. *Bio Protoc* **7**, doi:10.21769/BioProtoc.2110 (2017).
